# Development and Validation of a Model to Quantify Injury Severity in Real Time

**DOI:** 10.1001/jamanetworkopen.2023.36196

**Published:** 2023-10-09

**Authors:** Jeff Choi, Edward B. Vendrow, Michael Moor, David A. Spain

**Affiliations:** 1 Department of Surgery, Stanford University, Stanford, California; 2 Department of Computer Science, Stanford University, Stanford, California

## Abstract

**Importance:**

Quantifying injury severity is integral to trauma care benchmarking, decision-making, and research, yet the most prevalent metric to quantify injury severity—Injury Severity Score (ISS)— is impractical to use in real time.

**Objective:**

To develop and validate a practical model that uses a limited number of injury patterns to quantify injury severity in real time through 3 intuitive outcomes.

**Design, Setting, and Participants:**

In this cohort study for prediction model development and validation, training, development, and internal validation cohorts comprised 223 545, 74 514, and 74 514 admission encounters, respectively, of adults (age ≥18 years) with a primary diagnosis of traumatic injury hospitalized more than 2 days (2017-2018 National Inpatient Sample). The external validation cohort comprised 3855 adults admitted to a level I trauma center who met criteria for the 2 highest of the institution’s 3 trauma activation levels.

**Main Outcomes and Measures:**

Three outcomes were hospital length of stay, probability of discharge disposition to a facility, and probability of inpatient mortality. The prediction performance metric for length of stay was mean absolute error. Prediction performance metrics for discharge disposition and inpatient mortality were average precision, precision, recall, specificity, F1 score, and area under the receiver operating characteristic curve (AUROC). Calibration was evaluated using calibration plots. Shapley addictive explanations analysis and bee swarm plots facilitated model explainability analysis.

**Results:**

The Length of Stay, Disposition, Mortality (LDM) Injury Index (the model) comprised a multitask deep learning model trained, developed, and internally validated on a data set of 372 573 traumatic injury encounters (mean [SD] age = 68.7 [19.3] years, 56.6% female). The model used 176 potential injuries to output 3 interpretable outcomes: the predicted hospital length of stay, probability of discharge to a facility, and probability of inpatient mortality. For the external validation set, the ISS predicted length of stay with mean absolute error was 4.16 (95% CI, 4.13-4.20) days. Compared with the ISS, the model had comparable external validation set discrimination performance (facility discharge AUROC: 0.67 [95% CI, 0.67-0.68] vs 0.65 [95% CI, 0.65-0.66]; recall: 0.59 [95% CI, 0.58-0.61] vs 0.59 [95% CI, 0.58-0.60]; specificity: 0.66 [95% CI, 0.66-0.66] vs 0.62 [95%CI, 0.60-0.63]; mortality AUROC: 0.83 [95% CI, 0.81-0.84] vs 0.82 [95% CI, 0.82-0.82]; recall: 0.74 [95% CI, 0.72-0.77] vs 0.75 [95% CI, 0.75-0.76]; specificity: 0.81 [95% CI, 0.81-0.81] vs 0.76 [95% CI, 0.75-0.77]). The model had excellent calibration for predicting facility discharge disposition, but overestimated inpatient mortality. Explainability analysis found the inputs influencing model predictions matched intuition.

**Conclusions and Relevance:**

In this cohort study using a limited number of injury patterns, the model quantified injury severity using 3 intuitive outcomes. Further study is required to evaluate the model at scale.

## Key Points

**Question:**

Can a practical, contemporary data-based real-time prediction model quantify injury severity as well as the Injury Severity Score (ISS)?

**Findings:**

In this prognostic study of a data set of 372 573 traumatic injury encounters, a practical tool was developed to quantify injury severity using 3 interpretable outcomes: hospital length of stay, disposition to a facility, and inpatient mortality. The multitask deep learning model had comparable discrimination performance with the ISS and could facilitate point-of-care use in addition to post hoc research.

**Meaning:**

Results of this study suggest that a practical and interpretable model, integrated within an intuitive website, offers potential to quantify injury severity in real time.

## Teaser

This cohort study assesses whether a practical model that uses a limited number of injury patterns would have comparable discrimination performance compared with the Injury Severity Score.

## Introduction

The global burden of traumatic injuries is increasing.^1^ Many institutional trauma registries and national databases capture patient injury severity using the Injury Severity Score (ISS). Developed in 1974 from a cohort of 2128 people who experienced motor vehicle crashes in Maryland,^2^ the ISS quantifies injury severity from 1 (least severe) to 75 (most severe). The ISS is integral to trauma care benchmarking and research, constituting criteria for level I trauma center verification in the US^3^ and essential descriptive metric or analytic covariate in trauma literature.

Despite the potential of an injury severity metric to inform clinical decision-making and patient expectation setting, the ISS is not available at the bedside: it is calculated using complex administrative codes, sometimes weeks after patient discharge. Several studies developed models with superior mortality discrimination compared with the ISS using the more prevalent *International Statistical Classification of Diseases and Related Health Problems, Tenth Revision (ICD-10)* diagnosis codes.^4,5^ However, injury *ICD-10* diagnosis codes —numbering several thousand—are also challenging to obtain in real time. The chasm between developing and implementing a practical injury severity metric persists.

We aimed to develop a practical model that quantifies injury severity in real time using 3 outcomes: predicted hospital length of stay, probability of discharge to a facility, and probability of inpatient mortality. The Length of Stay, Disposition, Mortality (LDM) Injury Index (the model) was developed using a nationally representative cohort, underwent internal and external validation, and comprises a limited number of input variables, interpretable output, and a practical web application to facilitate real-time use. We hypothesized the model would have comparable discrimination performance compared with the ISS.

## Methods

We followed the Transparent Reporting of a Multivariable Prediction Model for Individual Prognosis or Diagnosis (TRIPOD) reporting guideline for this prognostic study. The study was approved by the Stanford University Institutional Review Board. Informed consent was waived as the research involved no more than minimal risk.

### Data Source

This secondary analysis used the Agency for Healthcare Research and Quality 2017-2018 National Inpatient Sample (NIS). The NIS is the largest publicly available all-payer inpatient health care database in the US, comprising an approximately 20% stratified sample of all discharges (including both trauma and nontrauma-centers).^6^

Our study population comprised admission encounters of adults aged 18 years or older who were admitted with a primary diagnosis of traumatic injury (*ICD-10* diagnosis codes: S00-S99). To reflect patients for whom our model would be used, we included encounters with longer than 2 hospitalization days (ie, for patients with minor injuries discharged from the emergency department, or those with major injuries who do not survive to be hospitalized, injury severity quantification would offer little marginal benefit for clinical decision-making). We excluded encounters with missing or unknown outcome variables (length of stay, mortality, discharge disposition), transferred to another hospital (ie, unknown final outcome), or injury *ICD-10* diagnosis codes encoding unspecified injuries (eFigure in Supplement 1). The final study cohort was divided 60-20-20 into training, internal validation, and test sets using stratified sampling partitioned by mortality with absolute counts of 223 545, 74 514, and 74 514 respectively. We used Python 3.10 (Python Software Foundation), together with the Pandas software library version 1.5.3 to preprocess the data set.

### Input Variables

Model input variables comprised 176 injuries, consolidated from 4-character injury *ICD-10* diagnosis codes (eTable 1 in Supplement 1). We consolidated the number of input variables to facilitate model usability. We consolidated injuries according to an a priori decision to delineate injuries that are (1) specific enough that management or associated outcomes are reasonably distinct (eg, delineating concussion vs subarachnoid hemorrhage) and (2) general enough that a nonspecialist clinician could readily identify and classify the injury (eg, abducens nerve injury, along with others, were consolidated as *cranial nerve injuries*). We excluded superficial injuries of various body regions that are unlikely to be clinically consequential on their own (eg, abrasions).

### Model Architecture

The model is a multitask deep learning model (2 hidden layers with 256 neurons each, ReLU activation functions, 0.3 dropout ratio) that predicts 3 outcomes: length of stay, probability of discharge to a facility, and probability of inpatient mortality. Multitask models make simultaneous predictions for different tasks using a single model, using knowledge learned from related tasks to improve generalizability and performance.^7^

The model predicted logits for binary outcomes (facility discharge, mortality) and log-transformed length of stay. Binary focal cross entropy loss for binary outcomes and mean squared error loss for length of stay were averaged to compute the overall loss.

We trained the model using Adam optimization for 32 epochs with a batch size of 1024 and learning rate of 0.001, reduced 10-fold for the last 8 epochs to facilitate convergence. A hyperparameter sweep based on validation performance metrics optimized the model size and dropout rate. The final model (ie, hyperparameters) was chosen from the epoch with the lowest validation set loss. All models were trained using the PyTorch version 2.0.1, a Python package.

### Model Explainability

Black box algorithms that obfuscate understanding how predictions are made may impede clinical model adoption.^8^ We conducted Shapley additive explanations (SHAP) analysis^9^ to explore whether reasonable input variables drove model predictions. A widely adopted explainable artificial intelligence technique, SHAP analysis quantifies, ranks, and displays the importance of each input variable for making predictions. Bee swarm plots of SHAP values displayed the 10 injuries most influential in predicting each outcome as determined by the magnitude of their SHAP score.

### Statistical Analysis

#### Performance Metrics

For discharge to a facility and mortality, we report average precision, precision (positive predictive value), recall (sensitivity), specificity, F1 score, and area under the receiver operating characteristic curve (AUROC). eTable 2 in Supplement 1 defines these metrics. For length of stay, we report mean absolute error, stratified for patients hospitalized fewer than 7, 7 to 14, and longer than 14 days. We computed 95% CIs for each performance metric by training and measuring performance from 10 random train/validation/test data splits (PyTorch version 2.0.1; Python).

#### Calibration and Output Interpretation

We calibrated our model using Platt scaling to output interpretable results. Platt scaling takes the raw prediction scores from a model as input and fits a logistic regression model on top of these scores to convert them into calibrated probabilities. The model outputs 3 numbers: Lx (x = predicted hospital length of stay [days]); Dy (y = predicted probability of discharge to a facility); and Mz (z = predicted probability of inpatient mortality). For example, a patient with L5 D20 M6 would be predicted to have a 5-day-long hospitalization, 20% probability of discharge to a facility, and 6% probability of inpatient mortality.

#### External Validation

Our external validation set comprised 3855 adults aged 18 years or more admitted to our American College of Surgeons Committee on Trauma—verified level I trauma center between January 2017 and June 2021, and hospitalized for longer than 2 days. Our center is located in a suburban area. We included patients who met criteria for the 2 highest of our institution’s 3 trauma activation levels; these comprise patients seen in the resuscitation area by our trauma surgery service, while patients in the lowest trauma activation level are triaged by the emergency medicine team. We compared our model discrimination performance with that of the ISS (min-max normalized). Key variables were extracted from our trauma registry, which is compiled via manual medical record review by trained trauma registrars.

#### Secondary Analyses

We evaluated how age or baseline frailty may affect model prediction performance. The trauma frailty outcomes index (TROUT Index) is a validated model that quantifies frailty of patients with injury using *ICD-10* diagnosis codes on a 0 to 100 scale (frailty risk strata: low [0-19], medium [20-36], high [≥37]).^10^ A preplanned secondary analysis delineated the model performance for test set patients with low, medium, and high frailty risk.

To evaluate whether age or baseline frailty combined with injury severity would improve prediction performance, we evaluated test set performance of 2 alternative prediction models, one trained using TROUT Index scores in addition to the 176 injuries as input variables and the other using age in addition to the 176 injuries. To facilitate model implementation, we integrated the model within a web application.^11^

## Results

### Data Source

Among 372 573 encounters that met inclusion criteria, the mean (SD) length of stay was 5.5 (6.9) days, 217 724 (58.4%) were associated with discharge to a facility, and 6882 (1.8%) with inpatient mortality. The most common injuries were hip fractures (33.4%), multiple rib fractures (9.7%), subdural hematoma (9.0%), and pelvis fractures (7.8%; eTable 3 in Supplement 1). The mean (SD) age was 68.7 (19.3) years, and 56.6% were female.

The 3855 patients in our external validation cohort were hospitalized a mean (SD) of 5.3 (8.1) days. 1451 patients (37.6%) were discharged to a facility and 118 patients (3.1%) died during their hospitalization. The mean (SD) age was 59.7 [22.4] years, and 36.5% were female.

### Model Performance

Table 1 compares the model prediction performance with that of the ISS. Overall, discrimination performance (for binary outcomes: discharge to a facility and mortality) on the external validation set were comparable (facility discharge AUROC: 0.67 [95% CI, 0.67-0.68] vs 0.65 [95% CI, 0.65-0.66]; recall: 0.59 [95% CI, 0.58-0.61] vs 0.59 [95% CI, 0.58-0.60]; specificity: 0.66 [95% CI, 0.66-0.66] vs 0.62 [95%CI, 0.60-0.63]; mortality AUROC: 0.83 [95% CI, 0.81-0.84] vs 0.82 [95% CI, 0.82-0.82] ; recall: 0.74 [95% CI, 0.72-0.77] vs 0.75 [95% CI, 0.75-0.76]; specificity: 0.81 [95% CI, 0.81-0.81] vs 0.76 [95% CI, 0.75-0.77]). For patients hospitalized fewer than 7 days, the mean absolute error of length of stay predictions was 1.6 (95% CI, 1.6-1.6) days; for those hospitalized 7 to 14 days, 3.9 (95% CI, 3.9-3.9) days; and longer than 14 days, 16.9 (95% CI, 16.9-16.9 days).

**Table 1.** Prediction Performance of a Model to Quantify Injury Severity in Real Time (Test Set, External Validation Set) Compared With the ISS (External Validation Set)

Measure	Value (95% CI)^a^					
	Model				ISS, external validation set	
	Test set^b^^,^^c^		External validation set^b^^,^^d^		Discharge to a facility	Mortality (inpatient)
	Discharge to a facility	Mortality (inpatient)	Discharge to a facility	Mortality (inpatient)		
AUROC	0.71 (0.71-0.72)	0.77 (0.76-0.77)	0.65 (0.65-0.66)	0.82 (0.82-0.82)	0.67 (0.67-0.68)	0.83 (0.81-0.84)
Average precision	0.74 (0.74-0.74)	0.09 (0.09-0.10)	0.54 (0.53-0.54)	0.22 (0.21-0.23)	0.57 (0.56-0.58)	0.18 (0.15-0.20)
Precision (positive predictive value)	0.73 (0.73-0.74)	0.04 (0.04-0.05)	0.48 (0.47-0.49)	0.09 (0.09-0.09)	0.51 (0.50-0.52)	0.11 (0.10-0.12)
Recall (sensitivity)	0.65 (0.65-0.66)	0.67 (0.66-0.69)	0.59 (0.58-0.60)	0.75 (0.75-0.76)	0.59 (0.58-0.61)	0.74 (0.72-0.77)
Specificity	0.67 (0.66-0.67)	0.72 (0.70-0.73)	0.62 (0.60-0.63)	0.76 (0.75-0.77)	0.66 (0.66-0.66)	0.81 (0.81-0.81)
F1 score	0.69 (0.69-0.69)	0.08 (0.08-0.08)	0.53 (0.52-0.54)	0.16 (0.16-0.16)	0.55 (0.54-0.56)	0.19 (0.18-0.21)

Abbreviations: AUROC, area under the receiver operating characteristics curve; ISS, Injury Severity Score.

^a^ Computed from 10 random model initializations.

^b^ Length of stay prediction could not be made using ISS, a scalar metric, alone.

^c^ For the test set, the ISS predicted length of stay with mean absolute error was 3.32 (95% CI, 3.30-3.34) days.

^d^ For the external validation set, the ISS predicted length of stay with mean absolute error was 4.16 (95% CI, 4.13-4.20) days.

### Calibration

**Figure 1.**
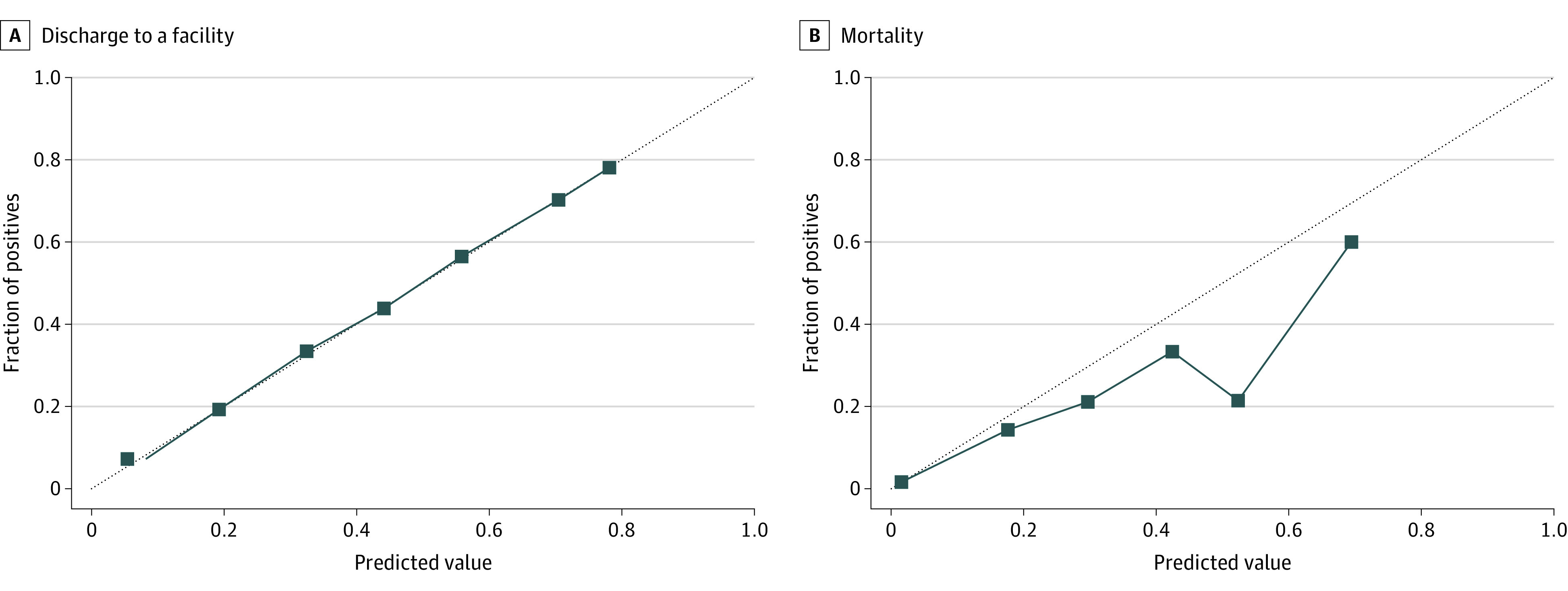
Injury Index Calibration for Prediction in the Test Set A perfect diagonal gray dotted line indicates perfect model calibration.

Figure 1 displays calibration plots for test set discharge disposition and mortality predictions. The model had strong calibration for predicting facility disposition, but overestimated mortality probability estimates.

### Model Explainability

**Figure 2.**
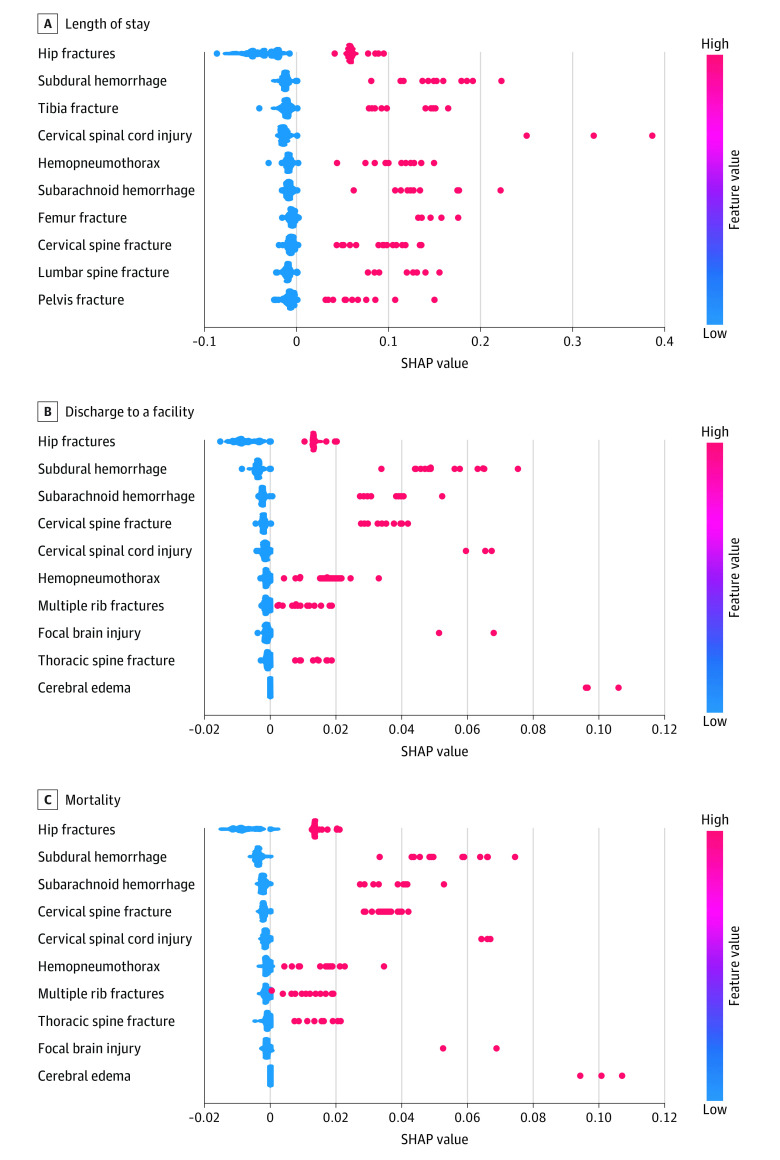
SHAP Values of the 10 Most Influential Features Associated With Predicting Length of Stay, Disposition, and Mortality Features are displayed in descending order of influence. Within each feature row, a dot represents the feature’s contribution to the prediction for an individual patient. The distribution of the dots for a feature provides a visual representation of the feature’s correlation with the prediction for the cohort. Positive SHAP (Shapley additive explanations) values have a positive impact on prediction (ie, longer length of stay, being discharged to a facility, or dying during hospitalization). Red dots with positive SHAP values indicate positive correlation between the input variable and prediction.

Figure 2 displays the 10 most influential injuries in predicting each outcome. The same injuries —many constituting hip fractures and traumatic brain or thoracic injuries— were associated with predicting discharge to a facility and mortality. More lower extremity fractures were influential in predicting length of stay.

### Secondary Analysis

Mean absolute errors of length of stay predictions were similar among patients with low, medium, and high frailty risk (Table 2). More patients with frailty had higher precision and lower recall performance for both discharge disposition and mortality. Compared with the model, an alternative prediction model including baseline frailty (TROUT Index scores) or age in addition to the 176 injuries as input variables had superior test set prediction performance for most outcomes (eTable 4 in Supplement 1).

**Table 2.** Model Prediction Performance on Test Set, Stratified by Baseline Frailty^a^

Measure	Value (95% CI)					
	Disposition to a facility			Mortality (inpatient)		
	Low	Medium	High	Low	Medium	High
AUROC	0.72 (0.72-0.72)	0.65 (0.65-0.65)	0.65 (0.65-0.65)	0.83 (0.82-0.84)	0.74 (0.73-0.75)	0.68 (0.67-0.68)
Average precision	0.69 (0.69-0.69)	0.81 (0.80-0.81)	0.84 (0.83-0.84)	0.097 (0.09-0.10)	0.13 (0.12-0.14)	0.10 (0.09-0.11)
Precision (positive predictive value)	0.67 (0.67-0.67)	0.82 (0.81-0.82)	0.85 (0.85-0.85)	0.04 (0.04-0.04)	0.06 (0.06-0.07)	0.08 (0.07-0.08)
Recall (sensitivity)	0.66 (0.66-0.66)	0.61 (0.60-0.61)	0.62 (0.61-0.63)	0.76 (0.75-0.76)	0.64 (0.62-0.65)	0.52 (0.49-0.55)
Specificity	0.66 (0.66-0.66)	0.65 (0.64-0.65)	0.65 (0.64-0.65)	0.76 (0.75-0.76)	0.74 (0.72-0.76)	0.74 (0.72-0.76)
F1 score	0.67 (0.66-0.67)	0.70 (0.69-0.70)	0.72 (0.71-0.72)	0.07 (0.07-0.07)	0.12 (0.11-0.12)	0.13 (0.12-0.14)

Abbreviation: AUROC, area under the receiver operating characteristic curve.

^a^ Frailty risk was calculated using the Trauma Frailty Outcomes Index scores, which quantifies frailty of injured patients on a scale of 0 to 100 (frailty risk: low [0-19], medium [20-36], high [≥37]). For length of stay with low frailty risk, the mean absolute error was 3.43 (95% CI, 3.42-3.45) days; with medium frailty risk, 3.08 (95% CI, 3.06-3.09) days; and with high frailty risk, 3.13 (95% CI, 3.10-3.16) days.

### Web Application

**Figure 3.**
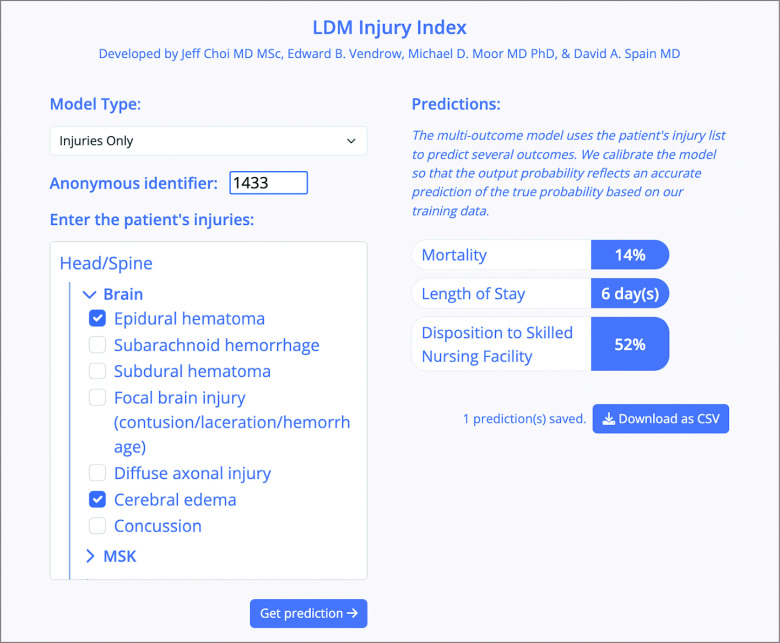
LDM Injury Index Website User Interface Users can select injury patterns (organized by body region and intuitive dropdown options) that match patterns of their patients and obtain predictions for 3 key outcomes in real time. Users can select a model that includes injury variables alone or an alternative model that uses age (years) or the TROUT (trauma frailty outcomes) Index score (marker of frailty) as an additional input variable. To support post hoc research, multiple patients’ injury pattern inputs and outputs (linked via anonymous identifiers) can be downloaded as a CSV file. LDM indicates length of stay, disposition, mortality. The model is available online.^11^

Users can navigate an intuitive website to input their patients’ injuries and see predictions in real time (Figure 3). Among 176 potential injuries, the relevant injuries can be easily selected using a web interface with a dropdown menu that organizes injuries by body region and type (eg, in abdomen/pelvis region, options include vascular, solid organ, genitourinary, musculoskeletal injuries). Moreover, if desired, users can use a toggle button to output predictions with an additional input variable: age (in years) or frailty (quantified by TROUT Index score). To facilitate further external validation and post hoc analysis, the website allows users to input multiple patients’ injury patterns and export predictions (along with selected injuries) as a CSV file.

## Discussion

We developed and validated the model, a practical model that quantifies injury severity using 3 interpretable outcomes. Despite using only 176 potential injuries as input variables, the model has a comparable discrimination performance with the ISS. Calibration for prediction discharge to a facility was excellent, but our model overestimated inpatient mortality. Explainability analysis found predictions were sensible. We developed an intuitive website to facilitate further model validation and future adoption as a point-of-care injury severity quantification metric.

Injury severity is not the sole determinant of patient outcomes. Corollary determinants include baseline frailty,^10,12,13^ time to receiving care,^14^ care facility,^15,16^ and less measurable outcomes such as quality of care.^17^ The ability of any injury severity quantification metric alone to predict outcomes has inherent limits. As expected, an alternative model with baseline frailty as an additional input variable increased all prediction performance metrics. The model lower recall and higher precision performance for patients with fragility also aligns with intuition. Given the same injury burden, underlying comorbidities—rather than the injury—may attribute to a higher proportion of mortalities among patients with fragility compared with patients with better health (ie, lower recall). On the other hand, a higher proportion of patients with fragility predicted to die actually died, as limited baseline physiologic reserve may decrease the chance of overcoming injury burden (ie, higher precision).

Despite inherent limitations of injury severity for predicting clinical outcomes, an injury severity quantification metric is important for benchmarking trauma care, standardizing vocabulary among clinicians, facilitating comparative research, and setting expectations with families. The ISS, ranging from 1 to 75, requires calculating Abbreviated Injury Scale (AIS) scores for each injury (ranging from 1 [minor] to 6 [nonsurvivable]) using a coding book (maintained by the Association for the Advancement of Automotive Medicine), and summing the squares of the 3 body regions with the highest AIS scores. Calculating the ISS using AIS scores requires specialized coding experts who review hospitalization records after discharge. Other prevalent injury severity metrics in trauma literature (the New ISS,^18^ the Trauma ISS,^19^ International Classification of Diseases ISS score^20^) noted prediction performance improvements but require complex input variables or formulas that hinder point-of-care use (eTable 5 in Supplement 1^21,22^). The Trauma ISS and International Classification of Diseases ISS Score output survival probabilities (the ISS and New ISS do not output clinically translatable outputs, beyond a higher number predicting worse outcomes), yet these reflect outcomes of injured patients from several decades ago.

With an accessible national trauma registry, higher computing power, and contemporary algorithms, several studies have explored using *ICD-10* diagnosis codes to predict mortality.^4,5^ Beyond higher discrimination and calibration performance compared with the ISS, these models using *ICD-10* diagnosis codes offer the added advantage of circumventing complex AIS codes and facilitating retrospective validation using variables recorded in most contemporary databases. However, the need to obtain *ICD-10* diagnosis codes and run algorithms without a dedicated user interface hinder point-of-care usability; predicting mortality retrospectively limits clinical utility.

To cross the model development-implementation chasm, clinical decision support models must be developed with end user implementation in mind. First, model inputs should comprise a limited number of readily obtainable variables. Reducing data dimensions could limit model performance, but this cost must be judge against the potential benefit of improved model usability. The model input variables comprise an intuitive list of 176 injuries: relevant injuries can be easily selected using a web interface with a dropdown menu that organizes injuries by body region and type.

Second, the algorithm must be appropriate for data set dimensions and consider the flexibility-interpretability tradeoff. Flexible algorithms (eg, deep neural networks) risk overfitting when trained on small data sets and are less interpretable (eg, multivariable logistic regression, a comparatively inflexible algorithm, yields interpretable odds ratios). Relative to input dimensions, the model was trained using a large data set, and external validation did not find evidence of overfitting. We implemented a model-agnostic explainability technique to ensure reasonable inputs informed outcome predictions.

Third, predicting clinical outcomes constitutes outcome-reasoning and must satisfy the MARA problem features.^8^ The model must be able to measure whether predictions match actual outcomes (measurement), change on a useful timescale (eg, in response to data shift; adaptable), tolerate prediction errors (resilient*)*, and tolerate potential incompatibility with user beliefs (agnostic). The model can store predictions for comparison against observed outcomes; can be updated using contemporary data; tolerate error because predictions do not mandate specific interventions; and tolerate user belief incompatibility because the singular or additive impact of injuries on outcomes is challenging to intuit.

Last, predicted outcomes must be interpretable and useful. In addition to mortality, the model outputs interpretable predictions for the probability of discharge to a facility and hospital length of stay, distinct metrics that may be important for clinicians and patients. We used multitask learning to accelerate model learning, reduce overfitting, and account for competing risks (ie, a patient who dies would not be at risk for facility disposition). Depending on clinical context or research question, different aspects of the 3 model outputs could be amplified.

### Limitations

Our study has several limitations. First, the list of injuries alone has inherent limitations in predicting outcomes of injured patients. Including noninjury input variables could improve outcome prediction performance yet obviate model independence as an injury severity metric for benchmarking or research applications. Similar to how the ISS is used as a covariate for inference studies or an input variable for predictive studies, we hope the model outputs (L, D, and M scores) can inform future studies as markers of injury burden. Second, the model does not include an exhaustive list of potential injuries as input variables. We excluded rare injuries to mitigate overfitting and reduce input variable dimensions; the bias directionality of patients with injuries not captured by our model is unknown. Third, interpreting the model outputs requires caution. For example, that 40% of patients with a particular injury pattern in a nationally representative cohort experienced inpatient mortality may not translate to a specific individual with the injury pattern having 40% chance of inpatient mortality (ecological fallacy). Fourth, our external validation cohort did not include patients with the lowest injury burden seen at our institution who met criteria for the lowest of our 3 trauma activation levels. Some performance decreases may be associated with the external validation cohort having higher injury burden compared with the NIS data set cohort (ie, higher mortality rate). Last, implementing the model at scale requires extensive study. The decision support systems driven by artificial intelligence (DECIDE-AI) guideline outlines critical steps for implementing clinical decision support models.^23^ For example, the model requires evaluation within diverse populations (eg, trauma or nontrauma centers), guidance on how outcomes could guide actions, and governance mechanism for error monitoring. Moreover, outcomes such as disposition to a facility and length of stay are confounded by factors external to injury burden, such as insurance and availability of care clinicians. The model will require iterative refinement and calibration to ensure the general performance remains stable across variable populations, and in response to potential data shifts over time (eg, expected outcomes for specific injuries may change with clinical advances). The current study is the first of many steps required to disseminate a safe and useful clinical decision support model.

## Conclusions

Results of this cohort study suggest that the model is a practical and interpretable multitask deep learning model that quantifies injury severity and is accessible to the public using an intuitive website with features that facilitate both point-of-care injury quantification and post-hoc research. The model was developed from a contemporary nationally representative cohort, externally validated, and built with end-user implementation in mind. In addition to supporting post hoc research, the model has potential to be the point-of-care alternative to the ISS, but extensive study is required in accordance with DECIDE-AI guidelines to evaluate the model at scale.

## Notes

Supplement 1. **eFigure.** Flow Diagram Showing Derivation of Study Cohort From 2017-2018 National Inpatient Sample **eTable 1.** Injuries Consolidated From 4 or 5-Digit ICD-10 Diagnosis Codes **eTable 2.** Definition of Performance Metrics **eTable 3.** Most Common Injuries Among Study Cohort Encounters **eTable 4.** Performance of LDM Injury Index and Modified Models (With TROUT Index Score or Age as an Additional Input Variable) on the Test Set **eTable 5.** Input Variables, Formula, and Development Populations for Commonly-Reported Injury Severity Metrics in Trauma Literature

Supplement 2.
Data Sharing Statement
