# Supplementary material for: Development and Validation of a Model to Quantify Injury Severity in Real Time
Source: JAMA Netw Open. 2023 Oct 9;6(10):e2336196. doi: 10.1001/jamanetworkopen.2023.36196 (PMC10562944; doi:10.1001/jamanetworkopen.2023.36196)
Supplement: Supplement 1. — eFigure. Flow Diagram Showing Derivation of Study Cohort From 2017-2018 National Inpatient Sample eTable 1. Injuries Consolidated From 4 or 5-Digit ICD-10 Diagnosis Codes eTable 2. Definition of Performance Metrics eTable 3. Most Common Injuries Among Study Cohort Encounters eTable 4. Performance of LDM Injury Index and Modified Models (With TROUT Index Score or Age as an Additional Input Variable) on the Test Set eTable 5. Input Variables, Formula, and Development Populations for Commonly-Reported Injury Severity Metrics in Trauma Literature [file jamanetwopen-e2336196-s001.pdf]

# Supplemental Online Content

Choi J, Vendrow EB, Moor MD, Spain DA. Development and validation of a model to quantify injury severity in real time. *JAMA Netw Open.* 2023;6(10):e2336196. doi:10.1001/jamanetworkopen.2023.36196

**eFigure.** Flow Diagram Showing Derivation of Study Cohort From 2017-2018 National Inpatient Sample

**eTable 1.** Injuries Consolidated From 4 or 5-Digit ICD-10 Diagnosis Codes

**eTable 2.** Definition of Performance Metrics

**eTable 3.** Most Common Injuries Among Study Cohort Encounters

**eTable 4.** Performance of LDM Injury Index and Modified Models (With TROUT Index Score or Age as an Additional Input Variable) on the Test Set

**eTable 5.** Input Variables, Formula, and Development Populations for Commonly-Reported Injury Severity Metrics in Trauma Literature

This supplemental material has been provided by the authors to give readers additional information about their work.

© 2023 Choi J et al. *JAMA Network Open*.

**eFigure: Flow diagram showing derivation of study cohort from 2017-2018 National Inpatient Sample**

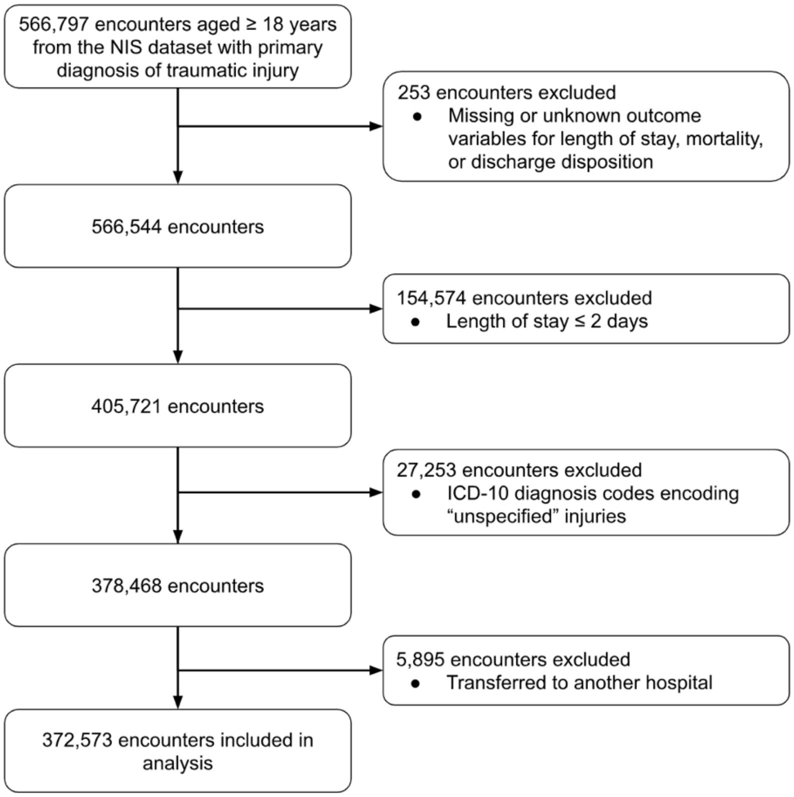


© 2023 Choi J et al. *JAMA Network Open*.

## eTable 1: Injuries consolidated from 4 or 5-digit ICD-10 diagnosis codes

| ICD-10 code | ICD-10 code injury description | Consolidated injury label |
|---|---|---|
| S35.0 | Injury of abdominal aorta | abdominal_aorta_art |
| S37.81 | Injury of the adrenal glands | adrenal |
| S98.0 | Traumatic amputation of foot at ankle level | amp_ankle_foot |
| S98.1 | Traumatic amputation of one toe | amp_ankle_foot |
| S98.2 | Traumatic amputation of two or more toes | amp_ankle_foot |
| S98.3 | Traumatic amputation of midfoot | amp_ankle_foot |
| S98.9 | Traumatic amputation of foot, level unspecified | amp_ankle_foot |
| S58.0 | Traumatic amputation at elbow level | amp_elbow_lower_arm |
| S58.1 | Traumatic amputation at level between elbow and wrist | amp_elbow_lower_arm |
| S58.9 | Traumatic amputation of forearm, level unspecified | amp_elbow_lower_arm |
| S38.2 | Traumatic amputation of external genital organs | amp_genitals |
| S68.0 | Traumatic amputation of thumb (complete)(partial) | amp_hand |
| S68.1 | Traumatic amputation of other single finger (complete)(partial) | amp_hand |
| S68.4 | Traumatic amputation of hand at wrist level | amp_hand |
| S68.5 | Traumatic transphalangeal amputation of thumb | amp_hand |
| S68.6 | Traumatic transphalangeal amputation of other and unspecified finger | amp_hand |
| S68.7 | Traumatic transmetacarpal amputation of hand | amp_hand |
| S78.0 | Traumatic amputation at hip joint | amp_hip_upper_leg |
| S78.1 | Traumatic amputation at level between hip and knee | amp_hip_upper_leg |
| S78.9 | Traumatic amputation of hip and thigh, level unspecified | amp_hip_upper_leg |
| S88.0 | Traumatic amputation at knee level | amp_knee_lower_leg |
| S88.1 | Traumatic amputation at level between knee and ankle | amp_knee_lower_leg |
| S88.9 | Traumatic amputation of lower leg, level unspecified | amp_knee_lower_leg |
| S08.1 | Traumatic amputation of ear | amp_part_head |
| S08.8 | Traumatic amputation of other parts of head | amp_part_head |
| S08.9 | Traumatic amputation of unspecified part of head | amp_part_head |
| S48.0 | Traumatic amputation at shoulder joint | amp_shoulder_upper_arm |
| S48.1 | Traumatic amputation at level between shoulder and elbow | amp_shoulder_upper_arm |
| S48.9 | Traumatic amputation of shoulder and upper arm, level unspecified | amp_shoulder_upper_arm |
| S38.3 | Transection (partial) of abdomen | amp_torso |
| S95.0 | Injury of dorsal artery of foot | art_foot |
| S95.1 | Injury of plantar artery of foot | art_foot |
| S08.0 | Avulsion of scalp | avulsion_scalp |
| S45.0 | Injury of axillary artery | ax_art |
| S45.2 | Injury of axillary or brachial vein | ax_brachial_vein |
| S37.2 | Injury of bladder | bladder |

© 2023 Choi J et al. *JAMA Network Open*.

| | | |
|---|---|---|
| S45.1 | Injury of brachial artery | brachial_art |
| S27.4 | Injury of bronchus | bronchus_trachea |
| S27.5 | Injury of thoracic trachea | bronchus_trachea |
| S26.0 | Injury of heart with haemopericardium | cardiac |
| S26.1 | Injury of heart without hemopericardium | cardiac |
| S26.9 | Injury of heart, unspecified with or without hemopericardium | cardiac |
| S15.0 | Injury of carotid artery | carotid_a |
| S35.2 | Injury of coeliac or mesenteric artery | celiac_sma_ima_art |
| S06.1 | Traumatic cerebral oedema | cerebral_edema |
| S36.5 | Injury of colon | colon |
| S06.0 | Concussion | concussion |
| S14.0 | Concussion and oedema of cervical spinal cord | cord_cspine |
| S14.1 | Other and unspecified injuries of cervical spinal cord | cord_cspine |
| S34.0 | Concussion and oedema of lumbar spinal cord | cord_lspine |
| S34.1 | Other injury of lumbar spinal cord | cord_lspine |
| S24.0 | Concussion and oedema of thoracic spinal cord | cord_tspine |
| S24.1 | Other and unspecified injuries of thoracic spinal cord | cord_tspine |
| S97.0 | Crushing injury of ankle | crush_ankle_foot |
| S97.1 | Crushing injury of toe(s) | crush_ankle_foot |
| S97.8 | Crushing injury of other parts of ankle and foot | crush_ankle_foot |
| S28.0 | Crushed chest | crush_chest |
| S38.0 | Crushing injury of external genital organs | crush_genitals |
| S67.0 | Crushing injury of thumb | crush_hand |
| S67.1 | Crushing injury of other and unspecified finger(s) | crush_hand |
| S67.2 | Crushing injury of hand | crush_hand |
| S67.3 | Crushing injury of wrist | crush_hand |
| S67.4 | Crushing injury of wrist and hand | crush_hand |
| S67.9 | Crushing injury of other and unspecified parts of wrist and hand | crush_hand |
| S07.0 | Crushing injury of Face | crush_head |
| S07.1 | Crushing injury of skull | crush_head |
| S07.8 | Crushing injury of other parts of head | crush_head |
| S07.9 | Crushing injury of head, part unspecified | crush_head |
| S87.0 | Crushing injury of knee | crush_knee_lower_leg |
| S87.8 | Crushing injury of other and unspecified parts of lower leg | crush_knee_lower_leg |
| S57.0 | Crushing injury of elbow | crush_lower_arm_elbow |
| S57.8 | Crushing injury of forearm | crush_lower_arm_elbow |
| S17.0 | Crushing injury of larynx and trachea | crush_neck |
| S17.8 | Crushing injury of other parts of Neck | crush_neck |
| S17.9 | Crushing injury of Neck, part unspecified | crush_neck |

© 2023 Choi J et al. *JAMA Network Open*.

| | | |
|---|---|---|
| S38.1 | Crushing injury of abdomen, lower back, and pelvis | crush_torso |
| S47.1 | Crushing injury of right shoulder and upper arm | crush_upper_arm |
| S47.2 | Crushing injury of left shoulder and upper arm | crush_upper_arm |
| S47.9 | Crushing injury of shoulder and upper arm, unspecified arm | crush_upper_arm |
| S77.0 | Crushing injury of hip | crush_upper_leg |
| S77.1 | Crushing injury of thigh | crush_upper_leg |
| S77.2 | Crushing injury of hip with thigh | crush_upper_leg |
| S27.80 | Diaphragm injury | diaphragm |
| S06.2 | Diffuse brain injury | diffuse_brain_injury |
| S33.0 | Traumatic rupture of lumbar intervertebral disc | disc_rupture_lspine |
| S23.0 | Traumatic rupture of thoracic intervertebral disc | disc_rupture_tspine |
| S93.0 | Dislocation of ankle joint | disloc_strain_sprain_foot |
| S93.1 | Dislocation of toe(s) | disloc_strain_sprain_foot |
| S93.3 | Dislocation of other and unspecified parts of foot | disloc_strain_sprain_foot |
| S93.4 | Sprain and strain of ankle | disloc_strain_sprain_foot |
| S93.5 | Sprain and strain of toe(s) | disloc_strain_sprain_foot |
| S93.6 | Sprain and strain of other and unspecified parts of foot | disloc_strain_sprain_foot |
| S63.0 | Subluxation and dislocation of wrist and hand joints | disloc_strain_sprain_hand |
| S63.1 | Subluxation and dislocation of thumb | disloc_strain_sprain_hand |
| S63.2 | Subluxation and dislocation of other finger(s) | disloc_strain_sprain_hand |
| S63.3 | Traumatic rupture of ligament of wrist and carpus | disloc_strain_sprain_hand |
| S63.4 | Traumatic rupture of ligament of finger at metacarpophalangeal and interphalangeal joint(s) | disloc_strain_sprain_hand |
| S63.5 | Sprain and strain of wrist | disloc_strain_sprain_hand |
| S63.6 | Sprain and strain of finger(s) | disloc_strain_sprain_hand |
| S63.8 | Sprain of other part of wrist and hand | disloc_strain_sprain_hand |
| S63.9 | Sprain of unspecified part of wrist and hand | disloc_strain_sprain_hand |
| S03.0 | Dislocation of jaw | disloc_strain_sprain_head |
| S03.1 | Dislocation of septal cartilage of nose | disloc_strain_sprain_head |
| S03.2 | Dislocation of tooth | disloc_strain_sprain_head |
| S03.3 | Dislocation of other and unspecified parts of head | disloc_strain_sprain_head |
| S03.4 | Sprain and strain of jaw | disloc_strain_sprain_head |
| S03.5 | Sprain and strain of joints and ligaments of other and unspecified parts of head | disloc_strain_sprain_head |
| S03.8 | Sprain and strain of joints and ligaments of other and unspecified parts of head | disloc_strain_sprain_head |
| S03.9 | Sprain and strain of joints and ligaments of other and unspecified parts of head | disloc_strain_sprain_head |
| S73.0 | Dislocation of hip | disloc_strain_sprain_hip |
| S73.1 | Sprain and strain of hip | disloc_strain_sprain_hip |
| S83.0 | Dislocation of patella | disloc_strain_sprain_knee |
| S83.1 | Dislocation of knee | disloc_strain_sprain_knee |

© 2023 Choi J et al. *JAMA Network Open*.

| | | |
|---|---|---|
| S83.2 | Tear of meniscus, current | disloc_strain_sprain_knee |
| S83.3 | Tear of articular cartilage of knee, current | disloc_strain_sprain_knee |
| S83.4 | Sprain and strain involving (fibular)(tibial) collateral ligament of knee | disloc_strain_sprain_knee |
| S83.5 | Sprain and strain involving (anterior)(posterior) cruciate ligament of knee | disloc_strain_sprain_knee |
| S83.6 | Sprain and strain of other and unspecified parts of knee | disloc_strain_sprain_knee |
| S83.8 | Sprain of other specified parts of knee | disloc_strain_sprain_knee |
| S83.9 | Sprain of unspecified site of knee | disloc_strain_sprain_knee |
| S53.0 | Dislocation of radial head | disloc_strain_sprain_lower_arm |
| S53.1 | Subluxation and dislocation of ulnohumeral joint | disloc_strain_sprain_lower_arm |
| S53.2 | Traumatic rupture of radial collateral ligament | disloc_strain_sprain_lower_arm |
| S53.3 | Traumatic rupture of ulnar collateral ligament | disloc_strain_sprain_lower_arm |
| S53.4 | Sprain and strain of elbow | disloc_strain_sprain_lower_arm |
| S33.1 | Subluxation and dislocation of lumbar vertebra | disloc_strain_sprain_lspine_pelvis |
| S33.2 | Dislocation of sacroiliac and sacrococcygeal joint | disloc_strain_sprain_lspine_pelvis |
| S33.3 | Dislocation of other and unspecified parts of lumbar spine and pelvis | disloc_strain_sprain_lspine_pelvis |
| S33.4 | Traumatic rupture of symphysis pubis | disloc_strain_sprain_lspine_pelvis |
| S33.5 | Sprain and strain of lumbar spine | disloc_strain_sprain_lspine_pelvis |
| S33.6 | Sprain and strain of sacroiliac joint | disloc_strain_sprain_lspine_pelvis |
| S33.8 | Sprain of other parts of lumbar spine and pelvis | disloc_strain_sprain_lspine_pelvis |
| S33.9 | Sprain of unspecified parts of lumbar spine and pelvis | disloc_strain_sprain_lspine_pelvis |
| S13.0 | Traumatic rupture of cervical intervertebral disc | disloc_strain_sprain_neck |
| S13.1 | Dislocation of cervical vertebra | disloc_strain_sprain_neck |
| S13.2 | Dislocation of other and unspecified parts of Neck | disloc_strain_sprain_neck |
| S13.4 | Sprain and strain of cervical spine | disloc_strain_sprain_neck |
| S13.5 | Sprain and strain of thyroid region | disloc_strain_sprain_neck |
| S13.8 | Sprain and strain of joints and ligaments of other parts of Neck | disloc_strain_sprain_neck |
| S13.9 | Sprain and strain of joints and ligaments of unspecified parts of Neck | disloc_strain_sprain_neck |
| S43.0 | Subluxation and dislocation of shoulder joint | disloc_strain_sprain_shoulder |
| S43.1 | Dislocation of acromioclavicular joint | disloc_strain_sprain_shoulder |
| S43.2 | Dislocation of sternoclavicular joint | disloc_strain_sprain_shoulder |
| S43.3 | Dislocation of other and unspecified parts of shoulder girdle | disloc_strain_sprain_shoulder |
| S43.4 | Sprain and strain of shoulder joint | disloc_strain_sprain_shoulder |
| S43.5 | Sprain and strain of acromioclavicular joint | disloc_strain_sprain_shoulder |

© 2023 Choi J et al. *JAMA Network Open*.

| | | |
|---|---|---|
| S43.6 | Sprain and strain of sternoclavicular joint | disloc_strain_sprain_shoulder |
| S43.8 | Sprain of other specified parts of shoulder girdle | disloc_strain_sprain_shoulder |
| S43.9 | Sprain of unspecified parts of shoulder girdle | disloc_strain_sprain_shoulder |
| S23.1 | Dislocation of thoracic vertebra | disloc_strain_sprain_thorax |
| S23.2 | Dislocation of other and unspecified parts of thorax | disloc_strain_sprain_thorax |
| S23.3 | Sprain and strain of thoracic spine | disloc_strain_sprain_thorax |
| S23.4 | Sprain and strain of ribs and sternum | disloc_strain_sprain_thorax |
| S23.5 | Sprain of unspecified parts of thorax | disloc_strain_sprain_thorax |
| S23.8 | Sprain of other specified parts of thorax | disloc_strain_sprain_thorax |
| S09.2 | Traumatic rupture of ear drum | eardrum_middle_inner_ear |
| S09.2 | Traumatic injury of middle and inner ear | eardrum_middle_inner_ear |
| S15.2 | Injury of external jugular vein | ejv |
| S06.4 | Epidural haemorrhage | epidural_hemorrhage |
| S27.81 | Injury of the esophagus | esophagus |
| S05.0 | Injury of conjunctiva and corneal abrasion without mention of foreign body | eye_orbit_injury |
| S05.1 | Contusion of Eyeball and orbital tissues | eye_orbit_injury |
| S05.2 | Ocular laceration and rupture with prolapse or loss of intraocular tissue | eye_orbit_injury |
| S05.3 | Ocular laceration without prolapse or loss of intraocular tissue | eye_orbit_injury |
| S05.4 | Penetrating wound of orbit with or without foreign body | eye_orbit_injury |
| S05.5 | Penetrating wound of Eyeball with foreign body | eye_orbit_injury |
| S05.6 | Penetrating wound of Eyeball without foreign body | eye_orbit_injury |
| S05.7 | Avulsion of Eye | eye_orbit_injury |
| S05.8 | Other injuries of Eye and orbit | eye_orbit_injury |
| S05.9 | Injury of Eye and orbit, part unspecified | eye_orbit_injury |
| S37.4 | Injury of ovary | female_reproductive |
| S37.5 | Injury of fallopian tube | female_reproductive |
| S37.6 | Injury of uterus | female_reproductive |
| S75.0 | Injury of femoral artery | femoral_art |
| S75.1 | Injury of femoral vein at hip and thigh level | femoral_vein |
| S22.5 | Flail chest | flail_chest |
| S06.3 | Focal brain injury | focal_brain_contusion_lac_ich |
| S42.0 | Fracture of clavicle | fx_clavicle |
| S12.0 | Fracture of first cervical vertebra | fx_cspine |
| S12.1 | Fracture of second cervical vertebra | fx_cspine |
| S12.2 | Fracture of third cervical vertebra | fx_cspine |
| S12.3 | Fracture of fourth cervical vertebra | fx_cspine |
| S12.4 | Fracture of fifth cervical vertebra | fx_cspine |
| S12.5 | Fracture of sixth cervical vertebra | fx_cspine |

© 2023 Choi J et al. *JAMA Network Open*.

| | | |
|---|---|---|
| S12.6 | Fracture of seventh cervical vertebra | fx_cspine |
| S72.3 | Fracture of shaft of femur | fx_femur |
| S72.4 | Fracture of lower end of femur | fx_femur |
| S72.8 | Fractures of other parts of femur | fx_femur |
| S72.9 | Fracture of femur, part unspecified | fx_femur |
| S79.2 | Physeal fracture of lower end of femur | fx_femur |
| S82.4 | Fracture of shaft of fibula | fx_fibula |
| S89.2 | Physeal fracture of upper end of fibula | fx_fibula |
| S89.3 | Physeal fracture of lower end of fibula | fx_fibula |
| S92.0 | Fracture of calcaneus | fx_foot |
| S92.1 | Fracture of talus | fx_foot |
| S92.2 | Fracture of other tarsal bone(s) | fx_foot |
| S92.3 | Fracture of metatarsal bone | fx_foot |
| S92.4 | Fracture of great toe | fx_foot |
| S92.5 | Fracture of other toe | fx_foot |
| S92.8 | Other fracture of foot, except ankle | fx_foot |
| S92.9 | Fracture of foot, unspecified | fx_foot |
| S99.0 | Physeal fracture of calcaneus | fx_foot |
| S99.1 | Physeal fracture of metatarsal | fx_foot |
| S99.2 | Physeal fracture of phalanx of toe | fx_foot |
| S62.0 | Fracture of navicular [scaphoid] bone of hand | fx_hand |
| S62.1 | Fracture of other carpal bone(s) | fx_hand |
| S62.2 | Fracture of first metacarpal bone | fx_hand |
| S62.3 | Fracture of other metacarpal bone | fx_hand |
| S62.5 | Fracture of thumb | fx_hand |
| S62.6 | Fracture of other finger | fx_hand |
| S62.9 | Unspecified fracture of wrist and hand | fx_hand |
| S72.0 | Fracture of head and Neck of femur | fx_hip |
| S72.1 | Pertrochanteric fracture | fx_hip |
| S72.2 | Subtrochanteric fracture | fx_hip |
| S79.0 | Physeal fracture of upper end of femur | fx_hip |
| S42.2 | Fracture of upper end of humerus | fx_humerus |
| S42.3 | Fracture of shaft of humerus | fx_humerus |
| S42.4 | Fracture of lower end of humerus | fx_humerus |
| S49.0 | Physeal fracture of upper end of humerus | fx_humerus |
| S49.1 | Physeal fracture of lower end of humerus | fx_humerus |
| S12.8 | Fracture of other parts of Neck | fx_hyoid_larynx_thyroid_trachea |
| S82.6 | Fracture of lateral malleolus | fx_lateral_mal |

© 2023 Choi J et al. *JAMA Network Open*.

| | | |
|---|---|---|
| S32.0 | Fracture of lumbar vertebra | fx_lspine |
| S02.4 | Fracture of malar and maxillary, zygoma bones | fx_malar_maxillary_zygoma |
| S82.84 | Bimalleolar fracture | fx_malleolus |
| S02.6 | Fracture of mandible | fx_mandible |
| S82.5 | Fracture of medial malleolus | fx_medial_mal |
| S02.2 | Fracture of nasal bones | fx_nasal |
| S02.3 | Fracture of orbital floor | fx_orbit |
| S82.0 | Fracture of patella | fx_patella |
| S32.3 | Fracture of ilium | fx_pelvis |
| S32.4 | Fracture of acetabulum | fx_pelvis |
| S32.5 | Fracture of pubis | fx_pelvis |
| S32.6 | Fracture of ischium | fx_pelvis |
| S32.8 | Fracture of other parts of pelvis | fx_pelvis |
| S52.1 | Fracture of upper end of radius | fx_radius |
| S52.3 | Fracture of shaft of radius | fx_radius |
| S52.5 | Fracture of lower end of radius | fx_radius |
| S59.1 | Physeal fracture of upper end of radius | fx_radius |
| S59.2 | Physeal fracture of lower end of radius | fx_radius |
| S22.3 | Fracture of one rib | fx_rib |
| S22.4 | Multiple fractures of ribs | fx_rib_multi |
| S32.1 | Fracture of sacrum | fx_sacrum_coccyx |
| S32.2 | Fracture of coccyx | fx_sacrum_coccyx |
| S42.1 | Fracture of scapula | fx_scapula |
| S42.9 | Fracture of shoulder girdle, part unspecified | fx_shoulder |
| S02.0 | Fracture of vault of skull | fx_skull |
| S02.1 | Fracture of base of skull | fx_skull |
| S22.2 | Fracture of sternum | fx_sternum |
| S82.1 | Fracture of upper end of tibia | fx_tibia |
| S82.2 | Fracture of shaft of tibia | fx_tibia |
| S82.3 | Fracture of lower end of tibia | fx_tibia |
| S89.0 | Physeal fracture of upper end of tibia | fx_tibia |
| S89.1 | Physeal fracture of lower end of tibia | fx_tibia |
| S02.5 | Fracture of tooth | fx_tooth |
| S22.0 | Fracture of thoracic vertebra | fx_tspine |
| S52.0 | Fracture of upper end of ulna | fx_ulna |
| S52.2 | Fracture of shaft of ulna | fx_ulna |
| S52.6 | Fracture of lower end of ulna | fx_ulna |
| S59.0 | Physeal fracture of lower end of ulna | fx_ulna |

© 2023 Choi J et al. *JAMA Network Open*.

| | | |
|---|---|---|
| S75.2 | Injury of greater saphenous vein at hip and thigh level | gsv_vein |
| S85.3 | Injury of greater saphenous vein at lower leg level | gsv_vein |
| S65.2 | Injury of superficial palmar arch | hand_art |
| S65.3 | Injury of deep palmar arch | hand_art |
| S65.4 | Injury of blood vessel(s) of thumb | hand_art |
| S65.5 | Injury of blood vessel(s) of other finger | hand_art |
| S27.0 | Traumatic pneumothorax | hptx |
| S27.1 | Traumatic haemothorax | hptx |
| S27.2 | Traumatic haemopneumothorax | hptx |
| S15.3 | Injury of internal jugular vein | ijv |
| S35.5 | Injury of iliac blood vessels | iliac_vessels |
| S25.1 | Injury of innominate or subclavian artery | innominate_subclavian_art |
| S25.3 | Injury of innominate or subclavian vein | innominate_subclavian_vein |
| S25.5 | Injury of intercostal blood vessels | intercostal_vessels |
| S06.81 | Injury of right internal carotid artery, intracranial portion | intracranial_ica_art_injury |
| S06.82 | Injury of left internal carotid artery, intracranial portion | intracranial_ica_art_injury |
| S35.1 | Injury of inferior vena cava | ivc_vein |
| S37.0 | Injury of kidney | kidney |
| S19.81 | Injury of larynx | larynx |
| S36.1 | Injury of liver or gallbladder or bile dict | liver_gb_bileduct |
| S85.4 | Injury of lesser saphenous vein at lower leg level | lsv_vein |
| S27.3 | Other injuries of lung | lung |
| S96.0 | Injury of muscle and tendon of long flexor muscle of toe at ankle and foot level | muscle_tendon_foot |
| S96.1 | Injury of muscle and tendon of long extensor muscle of toe at ankle and foot level | muscle_tendon_foot |
| S96.2 | Injury of intrinsic muscle and tendon at ankle and foot level | muscle_tendon_foot |
| S96.8 | Injury of other muscles and tendons at ankle and foot level | muscle_tendon_foot |
| S96.9 | Injury of unspecified muscle and tendon at ankle and foot level | muscle_tendon_foot |
| S66.0 | Injury of long flexor muscle and tendon of thumb at wrist and hand level | muscle_tendon_hand |
| S66.1 | Injury of flexor muscle and tendon of other finger at wrist and hand level | muscle_tendon_hand |
| S66.2 | Injury of extensor muscle and tendon of thumb at wrist and hand level | muscle_tendon_hand |
| S66.3 | Injury of extensor muscle and tendon of other finger at wrist and hand level | muscle_tendon_hand |
| S66.4 | Injury of intrinsic muscle and tendon of thumb at wrist and hand level | muscle_tendon_hand |
| S66.5 | Injury of intrinsic muscle and tendon of other finger at wrist and hand level | muscle_tendon_hand |
| S66.8 | Injury of other muscles and tendons at wrist and hand level | muscle_tendon_hand |
| S66.9 | Injury of unspecified muscle and tendon at wrist and hand level | muscle_tendon_hand |
| S09.1 | Injury of muscle and tendon of head | muscle_tendon_head |
| S56.0 | Injury of flexor muscle, fascia and tendon of thumb at forearm level | muscle_tendon_lower_arm |

© 2023 Choi J et al. *JAMA Network Open*.

| | | |
|---|---|---|
| S56.1 | Injury of flexor muscle, fascia and tendon of other and unspecified finger at forearm level | muscle_tendon_lower_arm |
| S56.2 | Injury of other flexor muscle, fascia and tendon at forearm level | muscle_tendon_lower_arm |
| S56.3 | Injury of extensor or abductor muscles, fascia and tendons of thumb at forearm level | muscle_tendon_lower_arm |
| S56.4 | Injury of extensor muscle, fascia and tendon of other and unspecified finger at forearm level | muscle_tendon_lower_arm |
| S56.5 | Injury of other extensor muscle, fascia and tendon at forearm level | muscle_tendon_lower_arm |
| S56.8 | Injury of other muscles, fascia and tendons at forearm level | muscle_tendon_lower_arm |
| S56.9 | Injury of unspecified muscles, fascia and tendons at forearm level | muscle_tendon_lower_arm |
| S86.0 | Injury of Achilles tendon | muscle_tendon_lower_leg |
| S86.1 | Injury of other muscle(s) and tendon(s) of posterior muscle group at lower leg level | muscle_tendon_lower_leg |
| S86.2 | Injury of muscle(s) and tendon(s) of anterior muscle group at lower leg level | muscle_tendon_lower_leg |
| S86.3 | Injury of muscle(s) and tendon(s) of peroneal muscle group at lower leg level | muscle_tendon_lower_leg |
| S86.8 | Injury of other muscles and tendons at lower leg level | muscle_tendon_lower_leg |
| S86.9 | Injury of unspecified muscle and tendon at lower leg level | muscle_tendon_lower_leg |
| S16.1 | Strain of muscle and tendon at Neck level | muscle_tendon_neck |
| S16.2 | Laceration of muscle and tendon at Neck level | muscle_tendon_neck |
| S16.8 | Other specified injury of of muscle and tendon at Neck level | muscle_tendon_neck |
| S16.9 | Unspecified injury of muscle and tendon at Neck level | muscle_tendon_neck |
| S29.0 | Injury of muscle and tendon at thorax level | muscle_tendon_thorax |
| S39.0 | Injury of muscle and tendon of abdomen, lower back and pelvis | muscle_tendon_torso |
| S46.0 | Injury of tendon of the rotator cuff of shoulder | muscle_tendon_upper_arm |
| S46.1 | Injury of muscle and tendon of long head of biceps | muscle_tendon_upper_arm |
| S46.2 | Injury of muscle and tendon of other parts of biceps | muscle_tendon_upper_arm |
| S46.3 | Injury of muscle and tendon of triceps | muscle_tendon_upper_arm |
| S46.8 | Injury of other muscles and tendons at shoulder and upper arm level | muscle_tendon_upper_arm |
| S46.9 | Injury of unspecified muscle and tendon at shoulder and upper arm level | muscle_tendon_upper_arm |
| S76.0 | Injury of muscle and tendon at hip and thigh level | muscle_tendon_upper_leg |
| S76.1 | Injury of quadriceps muscle and tendon | muscle_tendon_upper_leg |
| S76.2 | Injury of adductor muscle and tendon of thigh | muscle_tendon_upper_leg |
| S76.3 | Injury of muscle and tendon of the posterior muscle group at thigh level | muscle_tendon_upper_leg |
| S76.8 | Injury of other specified muscles, fascia and tendons at thigh level | muscle_tendon_upper_leg |
| S76.9 | Injury of unspecified muscles, fascia and tendons at thigh level | muscle_tendon_upper_leg |
| S14.3 | Injury of brachial plexus | nerve_brachial_plexus |
| S04.0 | Injury of optic Nerve and pathways | nerve_cranial |
| S04.1 | Injury of oculomotor Nerve | nerve_cranial |
| S04.2 | Injury of trochlear Nerve | nerve_cranial |
| S04.3 | Injury of trigeminal Nerve | nerve_cranial |
| S04.4 | Injury of abducent Nerve | nerve_cranial |

© 2023 Choi J et al. *JAMA Network Open*.

| | | |
|---|---|---|
| S04.5 | Injury of facial Nerve | nerve_cranial |
| S04.6 | Injury of acoustic Nerve | nerve_cranial |
| S04.7 | Injury of accessory Nerve | nerve_cranial |
| S04.8 | Injury of other cranial Nerves | nerve_cranial |
| S04.9 | Injury of unspecified cranial Nerve | nerve_cranial |
| S94.0 | Injury of lateral plantar Nerve | nerve_foot |
| S94.1 | Injury of medial plantar Nerve | nerve_foot |
| S94.2 | Injury of deep peroneal Nerve at ankle and foot level | nerve_foot |
| S94.3 | Injury of cutaneous sensory Nerve at ankle and foot level | nerve_foot |
| S94.8 | Injury of other Nerves at ankle and foot level | nerve_foot |
| S94.9 | Injury of unspecified Nerve at ankle and foot level | nerve_foot |
| S64.0 | Injury of ulnar Nerve at wrist and hand level | nerve_hand |
| S64.1 | Injury of median Nerve at wrist and hand level | nerve_hand |
| S64.2 | Injury of radial Nerve at wrist and hand level | nerve_hand |
| S64.3 | Injury of digital Nerve of thumb | nerve_hand |
| S64.4 | Injury of digital Nerve of other finger | nerve_hand |
| S64.8 | Injury of other Nerves at wrist and hand level | nerve_hand |
| S64.9 | Injury of unspecified Nerve at wrist and hand level | nerve_hand |
| S54.0 | Injury of ulnar Nerve at forearm level | nerve_lower_arm |
| S54.1 | Injury of median Nerve at forearm level | nerve_lower_arm |
| S54.2 | Injury of radial Nerve at forearm level | nerve_lower_arm |
| S54.3 | Injury of cutaneous sensory Nerve at forearm level | nerve_lower_arm |
| S54.8 | Injury of other Nerves at forearm level | nerve_lower_arm |
| S54.9 | Injury of unspecified Nerve at forearm level | nerve_lower_arm |
| S84.0 | Injury of tibial Nerve at lower leg level | nerve_lower_leg |
| S84.1 | Injury of peroneal Nerve at lower leg level | nerve_lower_leg |
| S84.2 | Injury of cutaneous sensory Nerve at lower leg level | nerve_lower_leg |
| S84.8 | Injury of other Nerves at lower leg level | nerve_lower_leg |
| S84.9 | Injury of unspecified Nerve at lower leg level | nerve_lower_leg |
| S34.2 | Injury of Nerve root of lumbar and sacral spine | nerve_lspine |
| S34.3 | Injury of cauda equina | nerve_lspine |
| S34.4 | Injury of lumbosacral plexus | nerve_lspine |
| S34.5 | Injury of lumbar, sacral and pelvic sympathetic nerves | nerve_lspine |
| S34.6 | Injury of peripheral Nerve(s) of abdomen, lower back and pelvis | nerve_lspine |
| S34.8 | Injury of other nerves at abdomen, lower back and pelvis level | nerve_lspine |
| S34.9 | Injury of unspecified nerves at abdomen, lower back and pelvis level | nerve_lspine |
| S14.4 | Injury of peripheral Nerves of Neck | nerve_other_neck |
| S14.5 | Injury of cervical sympathetic Nerves | nerve_other_neck |
| S14.8 | Injury of other Nerves of Neck | nerve_other_neck |

| | | |
|---|---|---|
| S14.2 | Injury of Nerve root of cervical spine | nerve_root_cspine |
| S24.3 | Injury of peripheral Nerves of thorax | nerve_tspine_other |
| S24.4 | Injury of thoracic sympathetic Nerves | nerve_tspine_other |
| S24.8 | Injury of other Nerves of thorax | nerve_tspine_other |
| S24.2 | Injury of Nerve root of thoracic spine | nerve_tspine_root |
| S44.0 | Injury of ulnar Nerve at upper arm level | nerve_upper_arm |
| S44.1 | Injury of median Nerve at upper arm level | nerve_upper_arm |
| S44.2 | Injury of radial Nerve at upper arm level | nerve_upper_arm |
| S44.3 | Injury of axillary Nerve | nerve_upper_arm |
| S44.4 | Injury of musculocutaneous Nerve | nerve_upper_arm |
| S44.5 | Injury of cutaneous sensory Nerve at shoulder and upper arm level | nerve_upper_arm |
| S44.7 | Injury of multiple Nerves at shoulder and upper arm level | nerve_upper_arm |
| S44.8 | Injury of other Nerves at shoulder and upper arm level | nerve_upper_arm |
| S44.9 | Injury of unspecified Nerve at shoulder and upper arm level | nerve_upper_arm |
| S74.0 | Injury of sciatic Nerve at hip and thigh level | nerve_upper_leg |
| S74.1 | Injury of femoral Nerve at hip and thigh level | nerve_upper_leg |
| S74.2 | Injury of cutaneous sensory Nerve at hip and thigh level | nerve_upper_leg |
| S74.8 | Injury of other Nerves at hip and thigh level | nerve_upper_leg |
| S74.9 | Injury of unspecified Nerve at hip and thigh level | nerve_upper_leg |
| S91.0 | Open wound of ankle | open_wound_foot |
| S91.1 | Open wound of toe(s) without damage to nail | open_wound_foot |
| S91.2 | Open wound of toe(s) with damage to nail | open_wound_foot |
| S91.3 | Open wound of other parts of foot | open_wound_foot |
| S61.0 | Open wound of finger(s) without damage to nail | open_wound_hand |
| S61.1 | Open wound of finger(s) with damage to nail | open_wound_hand |
| S61.2 | Open wound of other finger without damage to nail | open_wound_hand |
| S61.3 | Open wound of other finger with damage to nail | open_wound_hand |
| S61.4 | Open wound of hand | open_wound_hand |
| S61.5 | Open wound of wrist | open_wound_hand |
| S01.0 | Open wound of scalp | open_wound_head |
| S01.1 | Open wound of Eyelid and periocular area | open_wound_head |
| S01.2 | Open wound of nose | open_wound_head |
| S01.3 | Open wound of ear | open_wound_head |
| S01.4 | Open wound of cheek and temporomandibular area | open_wound_head |
| S01.5 | Open wound of lip and oral cavity | open_wound_head |
| S01.7 | Multiple open wounds of head | open_wound_head |
| S01.8 | Open wound of other parts of head | open_wound_head |
| S01.9 | Open wound of head, part unspecified | open_wound_head |
| S51.0 | Open wound of elbow | open_wound_lower_arm |

© 2023 Choi J et al. *JAMA Network Open*.

| | | |
|---|---|---|
| S51.8 | Open wound of other parts of forearm | open_wound_lower_arm |
| S81.0 | Open wound of knee | open_wound_lower_leg |
| S81.8 | Open wound of lower leg | open_wound_lower_leg |
| S11.0 | Open wound involving larynx and trachea | open_wound_neck |
| S11.1 | Open wound involving thyroid gland | open_wound_neck |
| S11.2 | Open wound involving pharynx and cervical oesophagus | open_wound_neck |
| S11.7 | Multiple open wounds of Neck | open_wound_neck |
| S11.8 | Open wound of other parts of Neck | open_wound_neck |
| S11.9 | Open wound of Neck, part unspecified | open_wound_neck |
| S21.0 | Open wound of breast | open_wound_thorax |
| S21.1 | Open wound of front wall of thorax without penetration into thoracic cavity | open_wound_thorax |
| S21.2 | Open wound of back wall of thorax without penetration into thoracic cavity | open_wound_thorax |
| S21.3 | Open wound of front wall of thorax with penetration into thoracic cavity | open_wound_thorax |
| S21.4 | Open wound of back wall of thorax with penetration into thoracic cavity | open_wound_thorax |
| S21.9 | Open wound of thorax, part unspecified | open_wound_thorax |
| S31.0 | Open wound of lower back and pelvis | open_wound_torso |
| S31.1 | Open wound of abdominal wall without penetration into peritoneal cavity | open_wound_torso |
| S31.2 | Open wound of penis | open_wound_torso |
| S31.3 | Open wound of scrotum and testes | open_wound_torso |
| S31.4 | Open wound of vagina and vulva | open_wound_torso |
| S31.5 | Open wound of unspecified external genital organs | open_wound_torso |
| S31.6 | Open wound of abdominal wall with penetration into peritoneal cavity | open_wound_torso |
| S31.8 | Open wound of other parts of abdomen, lower back and pelvis | open_wound_torso |
| S41.0 | Open wound of shoulder | open_wound_upper_arm |
| S41.1 | Open wound of upper arm | open_wound_upper_arm |
| S71.0 | Open wound of hip | open_wound_upper_leg |
| S71.1 | Open wound of thigh | open_wound_upper_leg |
| S79.8 | Unspecified injury of hip and thigh | Other specified injuries of hip and thigh |
| S09.0 | Injury of blood vessels of head, not elsewhere classified | other_intracranial_vessels |
| S15.8 | Injury of other blood vessels at Neck level | other_neck_vessels |
| S36.2 | Injury of pancreas | pancreas |
| S85.2 | Injury of peroneal artery | peroneal_art |
| S19.85 | Injury of pharynx or cervical esophagus | pharynx_cervical_eso |
| S27.6 | Injury of pleura | pleura |
| S85.0 | Injury of popliteal artery | popliteal_art |
| S85.5 | Injury of popliteal vein | popliteal_vein |
| S35.3 | Injury of portal or splenic vein | portal_splenic_vein |
| S37.82 | Injury of prostate | prostate |

© 2023 Choi J et al. *JAMA Network Open*.

| | | |
|---|---|---|
| S25.4 | Injury of pulmonary blood vessels | pulmonary_vessels |
| S55.1 | Injury of radial artery at forearm level | radial_art |
| S65.1 | Injury of radial artery at wrist and hand level | radial_art |
| S36.6 | Injury of rectum | rectum |
| S35.4 | Injury of renal blood vessels | renal_vessels |
| S36.4 | Injury of small intestine | small_bowel |
| S36.0 | Injury of spleen | spleen |
| S36.3 | Injury of stomach | stomach |
| S06.6 | Traumatic subarachnoid haemorrhage | subarachnoid_hemorrhage |
| S06.5 | Traumatic subdural haemorrhage | subdural_hemorrhage |
| S45.3 | Injury of superficial vein at shoulder and upper arm level | superficial_vein_upper_arm |
| S25.2 | Injury of superior vena cava | svc_vein |
| S25.0 | Injury of thoracic aorta | thoracic_aorta_art |
| S27.89 | Injutry of thymus or thoracic duct | thymus_thoracic_duct |
| S19.84 | Injutry of thyroid | thyroid |
| S85.1 | Injury of (anterior)(posterior) tibial artery | tib_art |
| S19.82 | Injury of trachea | trachea |
| S55.0 | Injury of ulnar artery at forearm level | ulnar_art |
| S65.0 | Injury of ulnar artery at wrist and hand level | ulnar_art |
| S37.1 | Injury of ureter | ureter |
| S37.3 | Injury of urethra | urethra |
| S95.2 | Injury of dorsal vein of foot | vein_foot |
| S55.2 | Injury of vein at forearm level | vein_lower_arm |
| S15.1 | Injury of vertebral artery | vetebral_a |
| S19.83 | Injury of vocal cord | vocal_cord |

© 2023 Choi J et al. *JAMA Network Open*.

**eTable 2: Definition of performance metrics**

| Metric | Explanation |
|---|---|
| Area under the receiver operating characteristic curve (AUROC) | Describes the probability that a randomly selected subject who had an outcome, would be "ranked" as having a higher probability of having the outcome than a randomly selected subject who did not have the outcome. Requires cautious interpretation within imbalanced datasets (large majority/small minority who experience an outcome); the value may be falsely inflated. The lowest possible AUROC (e.g. a random classifier) is 0.5. |
| Precision<br>(positive predictive value) | Among those who were predicted to have the outcome, how many actually had the outcome? Important metric when the cost of a false positive is high (e.g. if a major, irreversible intervention would be performed for those predicted to have an outcome) |
| Recall (sensitivity) | Among those who actually had the outcome, how many were predicted to have the outcome? Important metric when the cost of a false negative is high (e.g. models aiming to "screen" high risk patients) |
| Mean average precision | Average precision: weighted mean of precision values across multiple thresholds. A model typically outputs the probability of an outcome happening; a "threshold" probability [e.g. 0.5, 0. 68] then binarily classifies the output as "positive" [outcome happens] or "negative" [outcome does not happen]. The precision and recall values at various thresholds can be mapped on a precision-recall curve (a curve like the receiver operating characteristic curve, but more relevant for imbalanced datasets). The weight is the increase in recall from the previous threshold. The average precision can also be considered as a singular number to summarize the precision recall curve.<br><br>The mean average precision is the average of average precision for each outcome class. |
| F1 score | Harmonic mean of precision and recall |
| Specificity | Among those who actually did not have the outcome, how many were predicted not to have the outcome? |

© 2023 Choi J et al. *JAMA Network Open*.

**eTable 3: Most common injuries among study cohort encounters**

| Injury | No. (%) |
|---|---|
| Hip fracture | 124,575 (33.4%) |
| Multiple rib fractures | 36,206 (9.7%) |
| Subdural hematoma | 33,471 (9.0%) |
| Pelvic fracture | 28,985 (7.8%) |
| Open wound in the head | 27,170 (7.3%) |
| Tibial fracture | 26,062 (7.0%) |
| Hemopneumothorax | 23,749 (6.4%) |
| Lumbar spine fracture | 23,537 (6.3%) |
| Femur fracture | 21,757 (5.8%) |
| Subarachnoid hemorrhage | 20,680 (5.6%) |

© 2023 Choi J et al. *JAMA Network Open*.

**eTable 4. Performance of LDM Injury Index and modified models (with TROUT Index score or age as an additional input variable) on the test set.**

| | **Length of Stay** | | | **Discharge to a Facility** | | | **Mortality** | | |
|---|---|---|---|---|---|---|---|---|---|
| | Injury variables only | Injury + TROUT[a] | Injury + Age | Injury variables only | Injury + TROUT[a] | Injury + Age | Injury variable only | Injury + TROUT | Injury + Age |
| Mean absolute error, days | 3.32 [3.30-3.34] | 3.30 [3.28-3.32] | 3.30 [3.28-3.32] | -- | -- | -- | -- | -- | -- |
| AUROC | -- | -- | -- | 0.72 [0.71-0.72] | 0.75 [0.75-0.75] | 0.80 [0.80-0.80] | 0.76 [0.76-0.77] | 0.83 [0.82-0.83] | 0.79 [0.79-0.80] |
| Mean average precision | -- | -- | -- | 0.74 [0.74-0.74] | 0.78 [0.78-0.78] | 0.82 [0.82-0.82] | 0.09 [0.09-0.09] | 0.12 [0.11-0.12] | 0.10 [0.10-0.11] |
| Precision/Positive Predictive Value (PPV) | -- | -- | -- | 0.73 [0.73-0.74] | 0.75 [0.75-0.75] | 0.79 [0.78-0.79] | 0.04 [0.04-0.04] | 0.05 [0.05-0.05] | 0.05 [0.04-0.05] |
| Recall (Sensitivity) | -- | -- | -- | 0.66 [0.65-0.66] | 0.68 [0.68-0.68] | 0.72 [0.72-0.72] | 0.67 [0.65-0.68] | 0.75 [0.74-0.75] | 0.70 [0.69-0.72] |
| F1 score | -- | -- | -- | 0.69 [0.69-0.69] | 0.72 [0.71-0.72] | 0.75 [0.75-0.75] | 0.08 [0.08-0.08] | 0.10 [0.10-0.10] | 0.09 [0.08-0.09] |
| Negative Predictive Value (NPV) | -- | -- | -- | 0.58 [0.58-0.58] | 0.61 [0.60-0.61] | 0.65 [0.65-0.65] | 0.99 [0.99-0.99] | 0.99 [0.99-0.99] | 0.99 [0.99-0.99] |
| Specificity | -- | -- | -- | 0.67 [0.66-0.67] | 0.68 [0.68-0.69] | 0.72 [0.72-0.72] | 0.72 [0.70-0.73] | 0.75 [0.75-0.75] | 0.73 [0.71-0.74] |

Brackets denote 95% confidence intervals, computed from ten randomly bootstrapped data splits. *Abbreviations: AUROC= area under the receiver operating characteristics curve.*

[a]TROUT Index refers to the trauma frailty outcomes index[10]

© 2023 Choi J et al. *JAMA Network Open*.

## eTable 5. Input variables, formula, and development populations for commonly-reported injury severity metrics in trauma literature

| Metric | Required input variables | Formula | Development population |
|---|---|---|---|
| Injury Severity Score[2] | Abbreviated Injury Scale scores of each injury | $a^2 + b^2 + c^2$<br><br>a,b,c correspond to AIS scores of the three body regions with the worst injuries | 2,128 motor vehicle accident victims in Maryland<br>(1968-1969) |
| New Injury Severity Score[17] | Abbreviated Injury Scale scores of each injury | $a^2 + b^2 + c^2$<br><br>a,b,c correspond to AIS scores of the three worst injuries, regardless of body region | 6,585 injured patients at two level I trauma centers (1991-1994) |
| Trauma Injury Severity Score[18] | Abbreviated Injury Scale scores of each injury<br><br>Revised Trauma Score (Glasgow Coma Scale, systolic blood pressure, respiratory rate)[21]<br><br>Age Index (0 = <55 years old, 1 = ≥55 years old) | Survival probability = $1/(1+e^{-b})$ e<br><br>$b = b_0 + b_1$(Revised Trauma Score) + $b_2$(ISS) + $b_3$(ISS) | 80,544 injured patients across 139 North American hospitals[22] (1982-1987) |
| International Classification of Diseases Injury Severity Score[19] | ICD-9 injury diagnosis codes | Product of survival risk ratios for every injury<br><br>Survival risk ratios per ICD-9 injury diagnosis = number of times ICD-9 code seen in surviving patient / total number of ICD-9 code occurrence in North Carolina Hospital Discharge Registry (1990s) | 314,402 injured patients in the North Carolina Hospital Discharge Registry (1990-[b])<br><br>[b] *end year not specified; manuscript published in 1996* |

© 2023 Choi J et al. *JAMA Network Open*.
