# Supplementary material for: Development and Validation of a Model to Quantify Injury Severity in Real Time
Source: JAMA Netw Open. 2023 Oct 9;6(10):e2336196. doi: 10.1001/jamanetworkopen.2023.36196 (PMC10562944; doi:10.1001/jamanetworkopen.2023.36196)
Supplement: Supplement 2. — Data Sharing Statement [file jamanetwopen-e2336196-s002.pdf]

Data Sharing Statement

Choi. Development and Validation of a Model to Quantify Injury Severity in Real Time. *JAMA Netw Open*. Published October 06, 2023. doi:10.1001/jamanetworkopen.2023.36196

Data
Data available: No

Additional Information
Explanation for why data not available: Data, while available to all, must be purchase from HCUP. External validation data itself has PHI but we are happy to answer questions regarding analytic code
